# Global Rather Than Vertical‐Selective Saccadic Abnormalities in Progressive Supranuclear Palsy

**DOI:** 10.1002/acn3.70480

**Published:** 2026-07-07

**Authors:** Duy Duan Nguyen, Mark Paine, Soohyun Lee, Amir Fazlollahi, Baibin (Ben) Wang, John D. O'Sullivan, Peter J. Nestor

**Affiliations:** ^1^ Clem Jones Centre for Ageing Dementia Research, Queensland Brain Institute The University of Queensland St Lucia Queensland Australia; ^2^ Cognitive Health Program, Mater Research Institute The University of Queensland South Brisbane Queensland Australia; ^3^ University of Medicine and Pharmacy, Hue University Hue City Vietnam; ^4^ Neuro‐Ophthalmology and Neuro‐Otology Clinic, Department of Neurology Royal Brisbane & Women's Hospital Herston Queensland Australia; ^5^ Department of Neurology Royal Brisbane & Women's Hospital Herston Queensland Australia; ^6^ UQ Centre for Clinical Research, Faculty of Medicine The University of Queensland Herston Queensland Australia; ^7^ Department of Neurology Mater Adult Hospital, Mater Misericordiae Limited South Brisbane Queensland Australia

**Keywords:** eye movements, Lewy body disease, progressive supranuclear palsy, saccades

## Abstract

**Objective:**

To test whether vertical saccades are preferentially affected in Progressive Supranuclear Palsy (PSP).

**Methods:**

PSP patients (*n* = 24) were compared to age‐matched controls (*n* = 94) and two degenerative groups (Alzheimer's disease, *n* = 20; Lewy body disease, *n* = 50). Video‐oculography (sampling rate: 1000 Hz) was used to measure visually guided saccades (15.3° amplitude) in vertical and horizontal planes. Traditional corrected velocity alongside novel metrics of velocity interruption, vacillation, and directional instability were compared across diagnostic groups.

**Results:**

Vertical saccades were slower than horizontal in all groups including controls. Velocities were significantly slowed in PSP; however, the magnitude of slowing was comparable in vertical and horizontal directions. Similarly, vertical and horizontal velocities were strongly correlated in PSP (*r* = 0.77, *p* < 0.001), as they were in all other groups. Likewise, velocity interruption, vacillation, and directional instability were more disordered in the vertical compared to the horizontal axis in both controls and PSP (*p* < 0.05). Z‐scores of all metrics in PSP were abnormal but with no predilection for vertical saccades. Vacillation was a predictor of saccade velocity.

**Interpretation:**

Contrary to conventional belief, vertical saccades were not selectively impaired in PSP. Instead, the findings indicated a more global disruption of the saccadic system. The clinical observation of impaired vertical saccades in PSP appears to be because vertical saccades are slower and more disordered than horizontal in the general population; thus, when abnormalities of saccades develop in all directions, it first reaches a threshold to be noticeable at the bedside in the vertical plane.

## 1 Introduction

Saccadic eye movements are fundamental to visual exploration [1, 2]. They are controlled by a cortical–subcortical network converging on the superior colliculus, which relays signals to burst and omnipause neurons [1, 3]. Vertical saccades depend primarily on burst neurons within the rostral interstitial medial longitudinal fasciculus (riMLF) in the midbrain, whereas horizontal saccades are driven by the paramedian pontine reticular formation (PPRF) [2]. Both sets of burst neurons are gated by omnipause neurons in the nucleus raphe interpositus, which pause to permit saccade initiation [2, 4, 5].

Based on this anatomical segregation, preferential degeneration of the riMLF with relative sparing of the PPRF has been thought to underlie the early, prominent slowing of vertical saccades in progressive supranuclear palsy (PSP) [4]. The 1964 clinical description by Steele, Richardson and Olszewski noted vertical supranuclear palsy, with horizontal involvement in later stages [6]. It was subsequently highlighted that slowed saccades with normal range of movement could predate development of the gaze palsy [7]. Thus, vertical saccade slowing is a core feature in the Movement Disorder Society criteria for PSP [8] whereas horizontal saccades are thought to become abnormal at a later disease stage [5, 9]. Studies employing quantitative measurement—as opposed to bedside observation—that directly compared vertical and horizontal saccades are, however, scarce. Only one study reported vertical saccades being more afflicted than horizontal in a head‐to‐head comparison, but it did so with a limited sample size [5].

Saccades in PSP are not just slow, but also irregular, curved, and interrupted. Shaikh et al. described premature interruptions that stopped or diverted the eye mid‐saccade, producing misdirected trajectories [10], including the “round‐the‐houses” sign [11]. These abnormalities were attributed to maladaptive burst neuron firing and aberrant superior colliculus feedback [10]. Although these abnormalities were more prominent vertically, horizontal saccades have also been shown to be slowed, interrupted, and of reduced accuracy [5, 10, 12]. This raises the possibility of a generalized disruption of the saccadic system in PSP rather than one with a predilection for the vertical circuitry.

Thus, two non‐mutually exclusive hypotheses can be formulated. The first is one in which degeneration spreads sequentially from riMLF to PPRF. The second is one in which degeneration of a single anatomical locus/network explains saccadic abnormalities in both directions. The latter offers a more parsimonious account in terms of neurodegeneration, but then an explanation of the apparent predilection for vertical saccades is needed. Aside from informing the landscape of neurodegeneration in PSP, understanding saccade pathophysiology in more depth could pave the way for more objective biomarkers for both diagnosis and tracking change. To this end, it would also be desirable to develop methods that consider PSP patient frailty to minimize exclusions. For instance, the gold standard for saccade recording has been to measure with magnetic scleral search coils [2], numerous trials, and large‐amplitude saccades [5, 13]. In PSP, impaired blinking, blepharospasm, retrocollis, and behavioral issues hinder scleral coil use, lengthy protocols, and large‐amplitude paradigms. With these issues in mind, this study aimed to provide a deeper understanding of saccadic dysfunction in PSP and to do so with a non‐invasive, brief method that would be as well tolerated and inclusive of as many patients as possible.

## 2 Methods

We adhered to the STROBE checklist for cross‐sectional studies [14].

### 2.1 Participants

We prospectively recruited participants from September 2019 to February 2026. The patient groups were diagnosed by at least one of three experienced neurologists (PJN, JDO'S, or MP) following current diagnostic criteria. PSP patients were clinically diagnosed at probable level and subclassified based on the presenting and predominant clinical features following Movement Disorder Society 2017 criteria [8]. All PSP patients were followed up to ensure diagnostic certainty. Two other neurodegenerative diseases—Lewy body disease (LBD) and Alzheimer's disease—were included as disease control groups. The LBD group including Parkinson's disease, Parkinson's disease dementia, and Dementia with Lewy bodies were collapsed into the single group due to their common pathology and were diagnosed according to current diagnostic criteria for probable Parkinson's disease, or Dementia with Lewy bodies and Parkinson's disease dementia [15, 16, 17]. Clinically symptomatic AD with biomarker evidence of Alzheimer pathology (defined by either a positive amyloid‐PET scan or reduced amyloid‐β 1–42 and elevated phosphorylated tau/amyloid‐β 1–42 ratio in cerebrospinal fluid) was also included [18]. Age‐matched healthy controls were screened to ensure (1) no significant neurological or psychiatric dysfunction and (2) Addenbrooke's Cognitive Examination III (ACE‐III) > 88 [19]. All groups underwent global cognitive testing with the ACE‐III; patients with LBD and PSP were rated on the modified Hoehn and Yahr scale (H‐Y) [20]. The saccade recording was obtained at the first visit where probable PSP diagnostic criteria were met.

### 2.2 Eye Tracker Set‐Up

Monocular eye tracking was performed using the EyeLink 1000 system (SR Research Ltd., Mississauga, Canada) with a sampling rate of 1000 Hz. The device detects pupil and corneal reflection with infrared light. Participants were seated at a fixed distance of 100 cm from the centre of a 27‐in. monitor (Dell S2719DGF, 2560 × 1440 resolution) with heads stabilized using chin and forehead rests. The camera was positioned 50 cm in front of the participant and below the display screen. All recordings were performed in a completely darkened room to minimize external visual distractions. Built‐in algorithms provided by the manufacturer were used for the calibration and validation procedures. A 9‐point calibration and validation procedure within the range of ±7.9° horizontal and ±14.6° vertical were employed using static red‐dot targets at known eccentricities, with accepted mean fixation error < 1.0° and maximum error < 1.5° of visual angle. All participants were tested without corrective eyewear, and all were able to readily complete the calibration and validation.

### 2.3 Visual‐Guided Saccade Experiment

Saccadic eye movements were elicited by presenting a red target dot on a white background. The location of the dot switched between two eccentric locations of ±7.65° (horizontal) or ±7.65° (vertical), with a fixed display duration of 500 m‐seconds per target, thus only a single eccentricity (15.3°) was examined. Participants were reminded to fixate on the red dot as quickly and accurately as possible. Each participant completed first 10 horizontal trials (alternating between 5 leftward, 5 rightward) and then 10 vertical trials (alternating between 5 upward, 5 downward) with a brief break and drift correction between the two planes.

### 2.4 Data Analysis

All data processing was performed using MATLAB (MathWorks, Natick, MA). Saccades were automatically detected and segmented using a multi‐parameter algorithm adapted from the DEMoNS protocol [21], which has been validated on similar infrared oculography systems and designed for neurodegenerative populations. This multi‐parameter approach improves robustness against artifacts, is sensitive to slow saccade detection, and has good quality control [21, 22]. All detected saccades were included in the main‐sequence analysis because the amplitude—velocity relationship is considered to reflect brainstem burst neuron output across all saccade types [2, 23].

To understand qualitative profiles of saccadic movements, novel three‐dimensional plots were created to capture the pupil's position in horizontal, vertical, and time axes, enabling direct visualization of the trajectory of horizontal and vertical saccades in comparison to each other. These replaced traditional two‐dimensional plots that only depict either horizontal or vertical displacements versus time.

### 2.5 Velocity

To account for the inherent amplitude‐velocity relationship, we fitted the peak velocity following the ‘main sequence’ relationship [2]. We compared the candidate models (square‐root, power‐law, and exponential models) using Akaike Information Criterion with correction [24] and Bayesian Information Criterion [25]. This approach allows for a balanced fit and complexity, on horizontal and vertical traces separately. We found that the square root function had comparable performance to the two other fitting formulae given in the form of a single metric for each individual (Table S1A). Previous studies have shown that this fitting formula converged well with saccadic dynamics [26, 27].

The velocity metric was normalized to amplitude using one‐parameter root square function:

Peak velocity=Corrected velocity*√Amplitude

where Peak velocity is the saccade's peak velocity, amplitude is the corresponding amplitude (°) and the corrected velocity is the unitless scaling parameter [27].

### 2.6 Novel Velocity Interruption and Trajectory‐Based Metrics: Vacillation, Directional Instability

**FIGURE 1:**
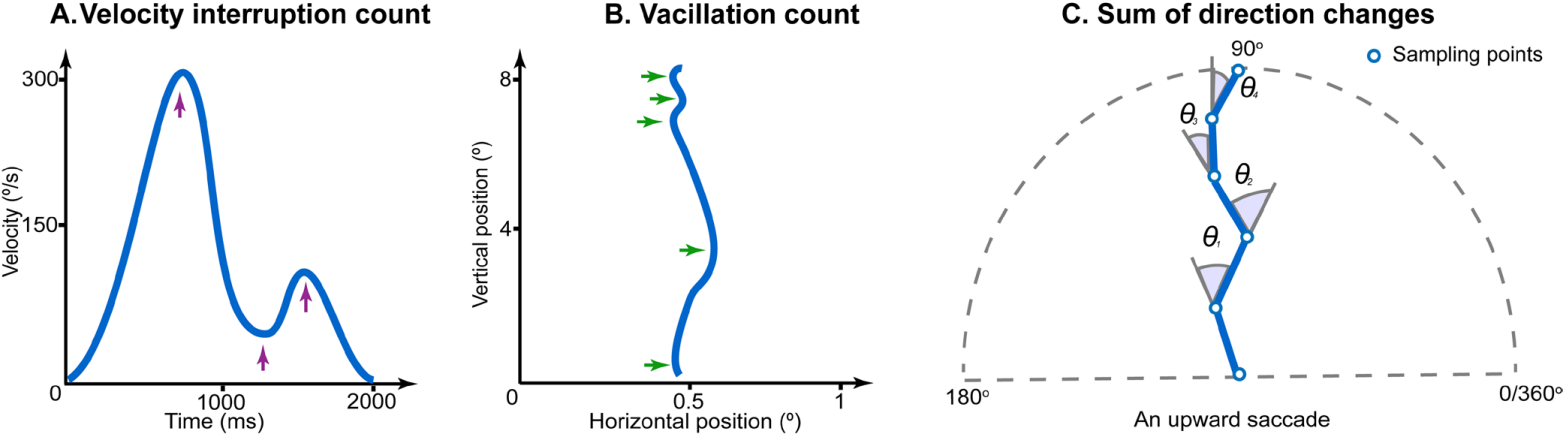
Novel saccade metrics: Velocity interruption count, vacillation count and sum of direction changes. (A) Velocity profile of a single saccade; each local peak or trough (magenta arrows) in the trace indicated a velocity interruption (in this example, the interruption count = 3). (B) Number of turning points (green arrows), illustrated here with an upward vertical saccade, corresponded to vacillation count (in this case, vacillation count = 5). (C) The sum of direction changes was the sum of absolute difference between two successive direction samples (in this example, the sum of direction changes = *θ*_1_ + *θ*_2_ + *θ*_3_ + *θ*_4_).

*Velocity interruption count* was defined as the number of local peaks and troughs in the velocity profile within a single saccade, capturing how often the eye alternates between acceleration and deceleration (i.e., how multiphasic the velocity profile is; Figure 1A, Method S1), as visualized in a previous study [10]. These irregular velocity fluctuations have been speculated to reflect brainstem and/or cerebellar output dysfunction [10, 13]. *Vacillation count* was defined as the number of turning points along the saccade flight that captures the irregularity of saccade trajectory (Figure 1B, Method S2). *Sum of direction changes* was defined as the sum of instantaneous direction differences between consecutive sampling points (Figure 1C, Method S3). Vacillation count and sum of directional change quantified the instability of online directional control along saccade excursion, which have been speculated to reflect superior colliculus dysfunction [10, 28, 29, 30].

Since all three metrics increased systematically with saccade amplitude, we expressed each as an amplitude‐normalized scaling parameter. For each metric, we fitted the raw values for each saccade against its corresponding amplitude using three simple one‐parameter functions (linear, square‐root, and log). Across metrics, the square‐root function consistently provided the best fit (lowest mean square error and Bayesian Information Criterion [25]; Table S1B–D), so analogous to corrected velocity, we adopted this model for the three novel metrics and the scaling parameters were called: velocity interruption, vacillation and directional instability, respectively.

### 2.7 Statistics

Data analyses followed a standardized statistical framework: normality was assessed using the Shapiro–Wilk test. Chi‐square test was used to compare sex ratios, and Wilcoxon signed‐rank tests were used for the non‐parametric H‐Y scale. Kruskal–Wallis with Dunn post hoc tests were employed to compare the medians of age, disease duration, and ACE‐III score.

Linear mixed‐effects models were built to compare velocity, velocity interruption, vacillation, and directional instability between fixed effects (i.e., diagnoses and directions) with a random intercept for each participant to account nested measures of vertical and horizontal in each participant. The covariates of age/sex were included in the model if they provided the best fit of the data using Anova function in R. All model assumptions were systematically verified. The sandwich method and cluster robust variance estimator (type 3) was used to estimate the *p*‐values and standard errors when homoskedasticity was violated as in vacillation and directional instability models. The post hoc estimated marginal means with Tukey correction was used to estimate the differences between diagnoses or directions. Considering the variation differences between vertical and horizontal in controls, exploratory Z‐score transformations were used to standardize PSP metrics using respective control distributions. Within the PSP group, vertical and horizontal z‐scores were compared using paired *t*‐test or Wilcoxon signed‐rank test and effect sizes were reported as Cohen's *d* or matched rank‐biserial correlation (*r*), respectively.

Pearson's correlation coefficients were computed to assess correlations between horizontal and vertical velocities and *p*‐values were corrected for multiple correlations using the Benjamini–Hochberg False Discovery Rate (FDR) procedure. The effect of diagnoses and interaction terms of diagnoses × directions in linear regression model tested whether diagnosis influenced the slope of correlation between vertical and horizontal velocities. Spearman's rank correlation to assess correlations between saccade parameters with disease severity (H‐Y scale or disease duration). PSP‐Richardson's syndrome and PSP‐non‐Richardson's syndrome were contrasted using Wilcoxon signed‐rank tests or *t*‐tests. A linear regression model was run to test whether the trajectory metrics or velocity interruption were predictors of velocity. All linear and mixed‐effects models are reported using unstandardized B coefficients. Statistical analyses were performed using R Studio version 4.4.3.

### 2.8 Ethics

The study was approved by the Mater Misericordiae Ltd. Human Ethics Committee (EC00332) and conducted in accordance with the local ethical guidelines. All participants gave informed consent prior to participation.

## 3 Results

### 3.1 Participants

25 probable PSP comprised *n* = 11 PSP‐Richardson's syndrome and *n* = 14 non‐Richardson's PSP phenotypes including PSP‐corticobasal syndrome (*n* = 5), PSP‐frontal (*n* = 4), PSP‐speech/language (*n* = 3), PSP‐progressive gait freezing (*n* = 1), and PSP‐parkinsonism (*n* = 1). All developed saccadic eye‐movement abnormalities and postural instability with longitudinal follow‐up (median follow up 12 [6–19] months). One PSP‐Richardson's syndrome was excluded because of complete vertical saccadic palsy meaning that no saccades were detectable. The final cohort therefore included 24 probable PSP patients. None of our PSP cohort was using Levodopa at the time saccades were recorded. One PSP‐CBS was under Amantadine. LBD patients were using Levodopa as regular medications.

### 3.2 Demographic Information

The LBD group was older than the AD and control groups, and male predominant. There were no significant differences in disease duration or global cognitive performance across diseased groups. There was no significant difference in H‐Y score between the PSP and LBD (see Table 1).

**TABLE 1:** Demographics and clinical characteristics of participants.

	Control (*n* = 94)	AD (*n* = 20)	LBD (*n* = 50)	PSP (*n* = 24)	Omnibus significance (*p*)
Age	64.9 ± 7.7	65.4 ± 8.4	71.5 ± 7.3	67.7 ± 7.4	< 0.001
Sex (M|F)	35|59	10|10	39|11	11|13	< 0.001
Disease duration (years)	—	3.0 ± 1.8	5.0 ± 4.8	3.5 ± 2.2	0.38
ACE‐III	94.7 ± 4.1	72.0 ± 14.4	83.9 ± 10.4	78.1 ± 13.5	< 0.001
H‐Y scale	—	—	1.8 ± 1.0	2.0 ± 1.6	0.20

*Note:* Post hoc comparisons: Age: control & AD < LBD (*p* < 0.05); Sex: LBD vs AD & PSP (*p* < 0.05); LBD vs control (*p* < 0.001); ACE‐III: control > AD, LBD, PSP (*p* < 0.001).

### 3.3 Three‐Dimensional Plots of Saccades

**FIGURE 2:**
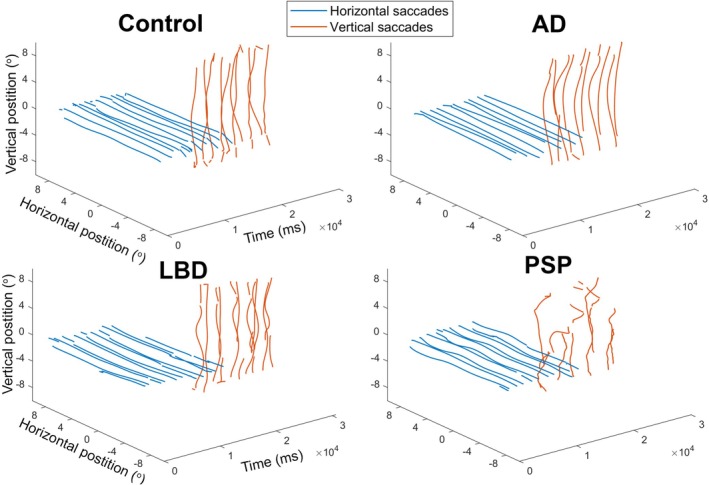
Three‐dimensional plots of saccade trajectories from four individual participants. Saccade trajectories plotted in horizontal, vertical, and time axes (ms). PSP saccades appeared to have frequent trajectory changes in horizontal and vertical planes, creating wobbly patterns. Small saccades in PSP could be remarkably deviated from the intended trajectory.

Three‐dimensional plots of saccades revealed that vertical saccades had more disordered trajectories than horizontal in all groups including healthy controls (examples of four individuals shown in Figure 2). Trajectories in both vertical and horizontal directions appeared wobblier and changed direction more frequently in PSP compared to the other groups.

### 3.4 Velocities of Horizontal and Vertical Saccades

**FIGURE 3:**
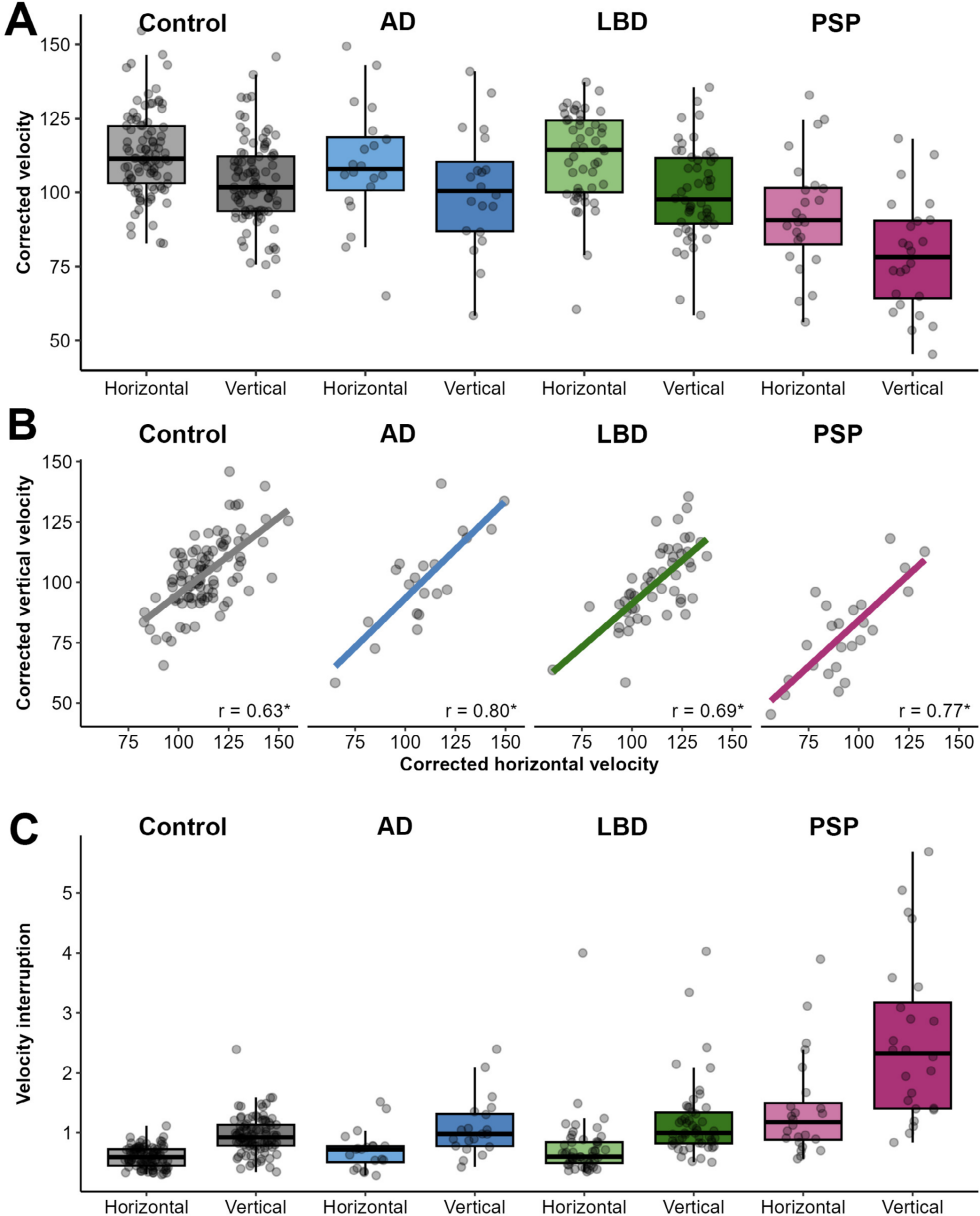
(A) Boxplot of corrected velocities across diagnoses and (B) Correlation of corrected vertical and horizontal velocities within groups (C) Boxplot of velocity interruptions across diagnoses. *p < 0.001.

The linear mixed model showed vertical saccades were significantly slower than horizontal in all groups, including controls (*p* < 0.005; see Figure 3A, Table S2A,B). In PSP, both vertical and horizontal velocities were significantly slower than other groups, including controls (*B* = 24.75 and 20.27 contrasting with controls, *p* < 0.001), while the velocities in AD and LBD were comparable to controls (*p* = NS, Table S2C). PSP z‐score for vertical and horizontal velocities was equally reduced (Cohen's *d* = 0.32, *p* = NS, Table S2D), indicating that the differences between vertical and horizontal velocities were similar within diagnostic groups. Similarly, there were moderate to strong positive correlations between vertical and horizontal velocities in all groups (Figure 3B) with no significant differences in the slope of the correlation across groups (*p* = NS, Table S3).

Regarding velocity interruption, there was a main effect of direction with vertical saccades being more interrupted than horizontal across all groups (*p* ≤ 0.001, Table S4A,B). The PSP velocity interruptions were significantly higher than others in both directions (*p* < 0.001, Table S4C). After Z‐transformation to account for the direction‐specific variances in controls, there was no significant difference in prevalence of velocity interruptions in horizontal versus vertical planes in PSP (*r* = 0.035, *p* = 0.66; Table S4D).

### 3.5 Novel Trajectory‐Based Metrics: Vacillation and Directional Instability

**FIGURE 4:**
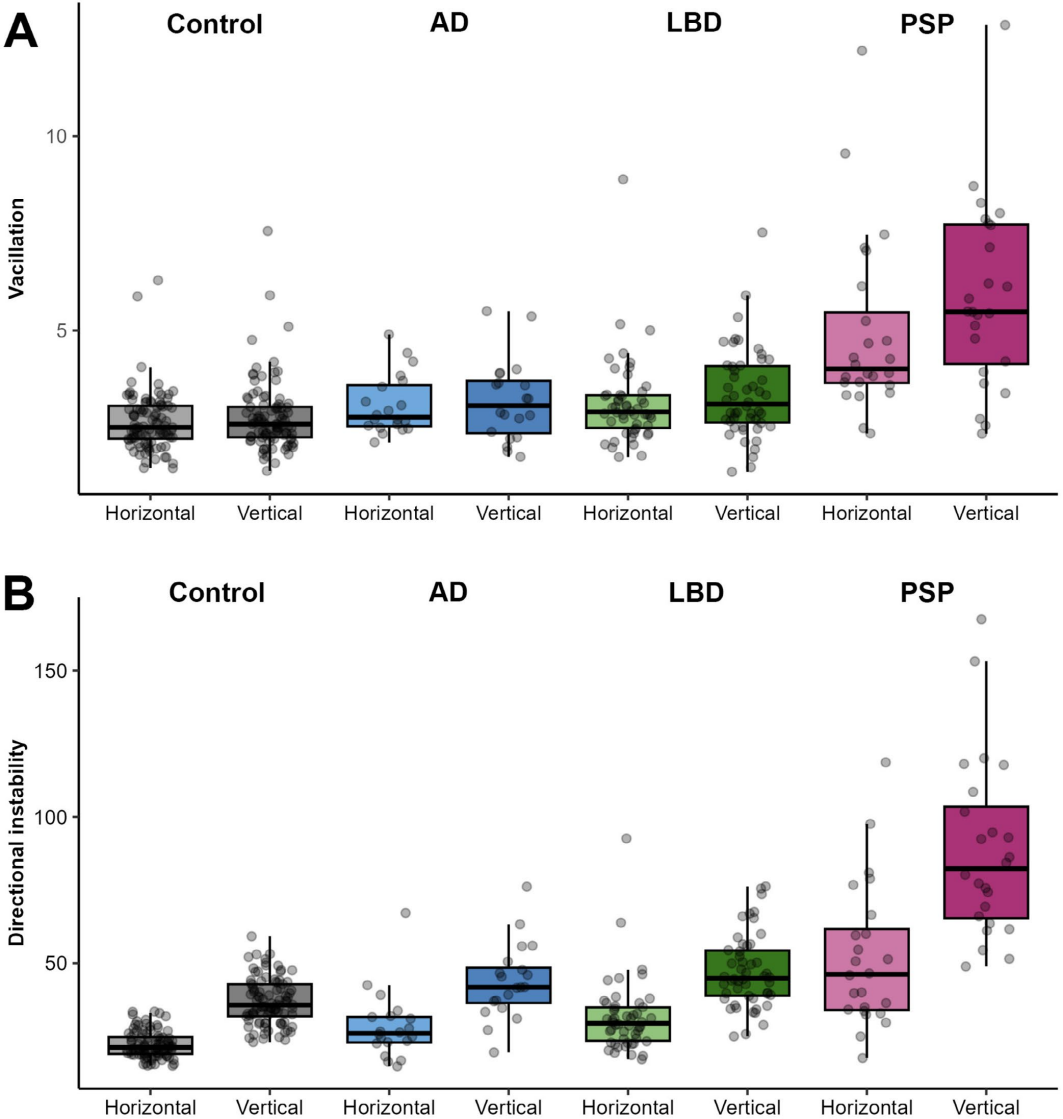
Boxplot of (A) vacillations and (B) directional errors of horizontal and vertical across groups.

A significant effect of direction was observed in the linear mixed model with vertical saccades showing more vacillations than horizontal in controls (*B* = 0.13, *p* = 0.04) and PSP (*B* = 0.96, *p* = 0.01) but not in the AD or LBD groups (*p* = NS, see Figure 4A and Table S5A–C). PSP z‐scores were similarly elevated in horizontal and vertical directions, indicating no evidence for a direction‐specific vacillation deficit (*r* = 0.175, *p* = NS, Table S5D).

There was a significant effect of age on directional instability, so mean‐centred age was included as a nuisance covariate in a linear mixed effect model. There was a main effect of direction, with vertical saccades being more directionally unstable than horizontal across all groups (*p* < 0.001, Table S6A–C). When directional instability was Z‐transformed, PSP horizontal and vertical z‐scores were comparably elevated (*r* = 0.023, *p* = 0.49, Table S6D).

### 3.6 Predictors of Slow Velocities in PSP

The linear regression model examining the relationship of trajectory‐based metrics to velocity in the PSP group indicated that the vacillation measure was a significant predictor of saccade velocity. Velocity was not significantly associated with directional instability measures. The was no significant interaction between vacillation and direction indicating that the association between vacillation and velocity was similar in horizontal and vertical directions (Table 2).

**TABLE 2:** Linear regression model examining trajectory metrics as predictors of saccade velocity in PSP.

Predictors	*B* coefficient	*p*	Adjusted *R* ^2^
Intercept	124.88	< 0.001	0.57
Direction vertical	−10.08	0.31	
Vacillation	−5.04	0.01	
Directional instability	−0.14	0.36	
Vacillation × Vertical	1.04	0.55	

### 3.7 Clinical Subtypes and Clinical Severity Correlation

PSP‐RS showed a numerical trend to greater impairment in all measures, compared to the non‐Richardson syndrome variants, however, none of these differences survived FDR correction for multiple comparisons (Table S7). No significant correlation was found between saccade metrics and disease staging (H‐Y score and disease duration, Table S8).

## 4 Discussion

The results indicated that vertical saccades were slower than horizontal in the general population. Once this was accounted for, slowing of saccades in PSP was comparable in both horizontal and vertical directions and they were strongly intercorrelated. Similarly, the number of interruptions was higher in the vertical direction across groups, and those of PSP showed significant increases in both directions.

Visualizing saccade trajectories as three‐dimensional plots with a 1000 Hz sampling rate enabled us to capture variability in saccade trajectories that have not been discernible from conventional two‐dimensional plots. These observations led to the development of novel trajectory‐based metrics: vacillation and directional instability. Both metrics were more disordered for vertical saccades compared to horizontal in controls. Z‐transforming the data to account for the difference between vertical and horizontal in controls, there were no significant differences in the degree to which these metrics were impaired in PSP in horizontal and vertical directions.

The findings demonstrated that vertical disadvantage, in terms of both velocity and accuracy of trajectory, is a general property of the oculomotor system. This could potentially be explained from an evolutionary perspective: vertical eye movement has been argued to be a recent development in response to bipedal posture and the manipulation of objects with the hands [31, 32]. Our results would support this narrative.

### 4.1 Vertical and Horizontal Velocities of Saccades in PSP Were Equally Impaired

The velocity corrected by “main sequence” is widely regarded as the behavioral proxy of burst neuron activity in the brainstem [2, 26]. Using this approach, Bhidayasiri et al. formally compared horizontal and vertical velocity reporting that vertical saccades were slower than horizontal in PSP [5]. From this, they concluded that the vertical burst generator—that is, riMLF—was preferentially afflicted in PSP. The study, however, only comprised seven PSP patients and seven controls; furthermore, it reported only qualitatively that vertical saccades were slowed compared to controls and that horizontal saccades were “less affected” but did not undertake any formal statistical analysis. Where the study did perform statistical analysis was in comparing vertical to horizontal saccades within the PSP group—finding that vertical saccades were significantly slower than horizontal. This was also true of the present study but only because there was a main effect of saccade direction with *all* groups showing slower vertical saccades than horizontal. At odds with the present study, Bhidayasiri et al.'s data suggested—albeit in only seven participants—that there was no difference in horizontal and vertical saccade velocities in healthy controls. This finding, however, appears anomalous in that other studies using the search coil technique have shown that vertical saccades were slower than horizontal in controls, in keeping with our findings [33, 34]. Furthermore, larger samples of healthy controls using video‐oculography to examine peak velocities, likewise demonstrated that horizontal saccades were intrinsically faster than vertical [35, 36]. Other studies of corrected velocity in PSP, that included both horizontal and vertical saccades, did not directly compare velocities of these two axes [10, 31, 37]. Several studies did not correct velocities for amplitude [35, 38, 39], meaning that slower velocity may have reflected smaller saccades rather than true changes in burst neuron activity [2]. Moreover, non‐corrected velocity is prone to be miscalculated due to potential gaze palsy in PSP [2]. Finally, other studies solely investigated either vertical [38] or horizontal saccades [12, 13, 40] in PSP. In summary, a critical review of past literature finds scant evidence for selective slowing of vertical saccades in PSP. Furthermore, pathological evidence of neuronal cell loss in the riMLF is lacking [4, 5, 41].

In a substantial control cohort (*N* = 94), the present study showed corrected velocities of vertical saccades to be significantly slower than horizontal. After accounting for this disparity, the magnitude of slowing in PSP was equivalent for horizontal and vertical saccades. Additionally, corrected vertical and horizontal velocities were tightly correlated in PSP, with the slope of the correlation remaining unchanged to other groups, indicating that slowing of saccades in both planes was occurring in concert. If preferential vertical involvement existed, one would observe divergence of the PSP regression line slope from that of the other groups. The results suggested a common underlying mechanism leading to reduction in velocity in both directions rather than from vertical‐specific pathology.

Our velocity interruption findings complement and extend those of Shaikh et al., who described that while velocity interruption in PSP was disordered in both directions, abnormalities were more pronounced in the vertical plane [10]. The study, however, did not explore whether the same disparity might also have been present in controls. By quantifying velocity interruption across all groups, we found that, similar to corrected velocity, interruptions occurred less frequently in the horizontal compared to the vertical axis in all groups. In agreement with Shaikh et al., we found the absolute frequency of velocity interruption was greater in the vertical plane in PSP; however, after accounting for control performance, there was no significant difference in the degree of abnormality observed with horizontal saccades.

Turning to the novel trajectory‐based metrics, only frequency of vacillation was a significant predictor of slow saccade velocity in PSP. Historically, the slowing of saccades in PSP has been attributed to the degeneration of burst neurons [4]. The relationship between vacillation and velocity in the present study suggests that there may be a common anatomical substrate for these two phenomena. The significant association with velocity suggests that vacillation may also contribute to the slowing of the saccade—that is, increased vacillation equates to longer saccade path in turn meaning a longer elapsed time to complete a given saccade amplitude. Alternatively, the result could be interpreted as vacillation being a product of slowed velocity (i.e., a slower velocity means more sampling points to observed vacillation). As with the other results, the relationship between vacillation and velocity was equally true of vertical and horizontal planes. Directional instability cannot, however, be considered a product of slowed velocity because there was no association between these two metrics. In non‐human primates, partial inhibition of superior colliculus activity has been reported to reduce saccade velocity and produce misdirected paths that require subsequent directional correction to reach the target [28, 29, 30]. In particular, it has been shown that inhibiting the superior colliculus leads to multiple changes in saccade trajectory [28] analogous to our vacillation and directional instability metrics.

Two other neurodegenerative diseases—LBD and AD—were included as disease control groups which do not primarily involve early degeneration of brainstem saccadic burst neuron circuits to the same extent as PSP. AD and LBD groups showed higher vacillation and directional instability compared to controls. The significant separation of PSP from AD and LBD across metrics suggests that the observed velocity and trajectory abnormalities were unlikely to reflect cortical neurodegeneration or cognitive impairment in general. Instead, they are more consistent with preferential involvement of the brainstem–cerebellar saccadic network in PSP.

In summary, the present results argue against a vertical saccade generator hypothesis in PSP [4] and instead point to a general dysfunction of saccade generation.

### 4.2 Clinical Implications

The finding that vertical and horizontal saccades are comparably impaired in PSP may appear at odds with conventional clinical dogma that vertical saccades are preferentially affected. This apparent contradiction can be reconciled by considering how abnormal saccades are defined at the bedside. A saccade is deemed abnormal if the observer can see its traverse whereas a normal saccade is so fast that the eye seems to instantaneously appear at each new location [8]. Because horizontal saccades are significantly faster than vertical in the general population, the threshold at which a saccade will become sufficiently slowed for an observer to see its traverse will be reached sooner in the vertical plane, in turn giving the illusion of preferential affliction of the vertical saccadic system. The same would apply for vacillation and directional instability.

Although there are several clinical presentations of PSP, abnormal saccadic movements represent a universal physiological feature [8] making quantifiable metrics thereof potential biomarkers for clinical trials. Despite the historical focus of vertical saccades, the present findings argue that horizontal saccades have greater biomarker potential for longitudinal studies, such as intervention trials. Vertical saccades reach “floor” earlier in the disease course and when this occurs longitudinal measurement of changes becomes impossible. For instance, in the current series one patient was excluded because of complete vertical gaze palsy, yet they still had recordable horizontal saccades.

Pooling PSP subtypes was intentional and aligned with the primary aim of the study, which was to characterize quantitative saccadic abnormalities across clinically established probable PSP as a spectrum, rather than to define subtype‐specific oculomotor signatures. Although the timing and prominence of oculomotor signs may differ between PSP phenotypes [2, 42], slow vertical saccades or supranuclear gaze palsy are core features of all probable PSP [8] and this criterion was met by all PSP patients in this study. PSP‐RS showed numerically greater impairment across several metrics, but these differences did not survive correction for multiple comparisons. Thus, the difference with respect to the oculomotor disorder was that it was present from an earlier disease stage in PSP‐RS compared to the other variants. It was, therefore, unsurprising that the oculomotor disorder was slightly, but not significantly, further evolved in the PSP‐RS group.

Strengths of the current study were the use of a simplified protocol with brief, ecological saccadic tasks [43, 44]—appropriate for frail and cognitively impaired patients—as well as the use of a square root function that performs reliably with relatively few saccades [26, 27].

There are some limitations to be addressed. Firstly, the main limitation of the study was the lack of pathological confirmation; however, all diagnoses we made by experienced neurologists using current Movement Disorder Society criteria and patients were followed longitudinally to ensure stability of diagnosis. Secondly, we acknowledge that video‐oculography is prone to miscalibration due to oculomotor palsy which means participants cannot foveate on target [2]. We believe that this issue is not a confound to our results for the following reasons: we calibrated and tested within relatively small amplitude in the vertical plane. This reduces the need for large vertical excursions, known to be impaired in PSP. Nonetheless, we did exclude one patient with complete vertical gaze palsy. Finally, in a previous simulated experiment, the relationship between peak velocity and amplitude (i.e., corrected velocity) was almost unchanged despite calibration error [2]. Nevertheless, residual calibration error cannot be completely excluded. Thirdly, our simplified saccadic protocol with relatively small number of trials may reduce the reliability of individual‐level measurements. We mitigated this limitation by (i) including all saccades (i.e., main, anticipatory, and corrective), comprising a range of amplitudes that could be fitted to the ‘main‐sequence’ formula and (ii) using a fitted square‐root function that has been shown to require fewer saccades to yield stable results [23]. Nonetheless, greater numbers of trials may have offered greater stability. Ultimately there is a trade‐off between measurement accuracy and feasibility in a clinically impaired PSP cohort. More arduous protocols would likely exclude patients, in turn, leading to a different form of unreliability.

Contrary to conventional belief, vertical saccades were not selectively impaired in PSP. The findings highlight a range of quantifiable metrics that could be valuated further for their potential to non‐invasively measure longitudinal change.

## Author Contributions

P.J.N., D.D.N., M.P., S.L., J.D.O.S. contributed to the conception and design of the study; P.J.N., S.L., D.D.N., A.F., B.B.W. contributed to the acquisition and analysis of data; D.D.N., P.J.N., B.B.W. contributed to the drafting a significant portion of the manuscript or figures.

## Funding

This work was supported by Mater Foundation; NHMRC Ideas grant 2023/GNT2030157.

## Conflicts of Interest

The authors declare no conflicts of interest.

## Supporting information


**Figure S1:** 3‐dimension plots of saccade trajectories from a further 8 individuals.
**Method S1:** Velocity interruption count.
**Method S2:** Vacillation count.
**Method S3:** Sum of direction changes.
**Table S1A:** Fitting performance of three most common used ‘main sequence’ fitting models for corrected velocity.
**Table S1B:** Fitting performance of three single‐parameter formula for velocity interruption.
**Table S1C:** Fitting performance of three single‐parameter formula for vacillation.
**Table S1D:** Fitting performance of three single‐parameter formula for directional instability.
**Table S2A:** Linear mixed model comparing the corrected velocity with fixed effects of diagnosis, direction and their interactions; with random intercept for each participant.
**Table S2B:** Contrast of estimated marginal means of corrected velocities within diagnoses.
**Table S2C:** Contrast of estimated marginal means of corrected velocities between diagnoses.
**Table S2D:** Direction‐specific z‐standardized corrected velocity in PSP.
**Table S3:** Linear model investigating the vertical and horizontal corrected velocity relationship across diagnoses.
**Table S4A:** Linear mixed model comparing the velocity interruption with fixed effects of diagnosis, direction and their interactions; with random intercept for each participant.
**Table S4B:** Contrast of estimated marginal means of vertical—horizontal velocity interruption within diagnoses.
**Table S4C:** Contrast of estimated marginal means of velocity interruption between diagnoses.
**Table S4D:** Direction‐specific z‐standardized velocity interruption in PSP.
**Table S5A:** Linear mixed effect model to predict vacillation basing on fixed effects: diagnosis, direction and their interaction and random effect: individual participant.
**Table S5B:** Contrast of estimated marginal means of vertical—horizontal vacillation within diagnoses.
**Table S5C:** Contrast of estimated marginal means of vacillation between diagnoses.
**Table S5D:** Direction‐specific z‐standardized vacillation in PSP.
**Table S6A:** Linear mixed model comparing the directional instability with fixed effects of diagnosis, direction and their interactions; with random intercept for each participant.
**Table S6B:** Contrast of estimated marginal means of vertical—horizontal directional instability within diagnoses.
**Table S6C:** Contrast of estimated marginal means of directional instability between diagnoses.
**Table S6D:** Direction‐specific z‐standardized of directional instability in PSP.
**Table S7:** Sensitivity analysis of PSP subgroup (PSP‐RS and PSP‐nonRS) cross metrics.
**Table S8:** Correlation table between disease stage and oculomotor metrics in PSP.

## Data Availability

The data that support the findings of this study are available from the corresponding author on reasonable request.
