# Supplementary material for: Global Rather Than Vertical‐Selective Saccadic Abnormalities in Progressive Supranuclear Palsy
Source: Ann Clin Transl Neurol. 2026 Jul 7:10.1002/acn3.70480. Online ahead of print. doi: 10.1002/acn3.70480 (PMC13394196; doi:10.1002/acn3.70480)
Supplement: Supplementary file 1 — Figure S1: 3‐dimension plots of saccade trajectories from a further 8 individuals. Method S1: Velocity interruption count. Method S2: Vacillation count. Method S3: Sum of direction changes. Table S1A: Fitting performance of three most common used ‘main sequence’ fitting models for corrected velocity. Table S1B: Fitting performance of three single‐parameter formula for velocity interruption. Table S1C: Fitting performance of three single‐parameter formula for vacillation. Table S1D: Fitting performance of three single‐parameter formula for directional instability. Table S2A: Linear mixed model comparing the corrected velocity with fixed effects of diagnosis, direction and their interactions; with random intercept for each participant. Table S2B: Contrast of estimated marginal means of corrected velocities within diagnoses. Table S2C: Contrast of estimated marginal means of corrected velocities between diagnoses. Table S2D: Direction‐specific z‐standardized corrected velocity in PSP. Table S3: Linear model investigating the vertical and horizontal corrected velocity relationship across diagnoses. Table S4A: Linear mixed model comparing the velocity interruption with fixed effects of diagnosis, direction and their interactions; with random intercept for each participant. Table S4B: Contrast of estimated marginal means of vertical—horizontal velocity interruption within diagnoses. Table S4C: Contrast of estimated marginal means of velocity interruption between diagnoses. Table S4D: Direction‐specific z‐standardized velocity interruption in PSP. Table S5A: Linear mixed effect model to predict vacillation basing on fixed effects: diagnosis, direction and their interaction and random effect: individual participant. Table S5B: Contrast of estimated marginal means of vertical—horizontal vacillation within diagnoses. Table S5C: Contrast of estimated marginal means of vacillation between diagnoses. Table S5D: Direction‐specific z‐standardized vacillation in PSP. Table S6A: Lin [file ACN3-9999-0-s001.docx]

**SUPPLEMENTARY**

**Figure S1. 3-dimension plots of saccade trajectories from a further 8 individuals**

**
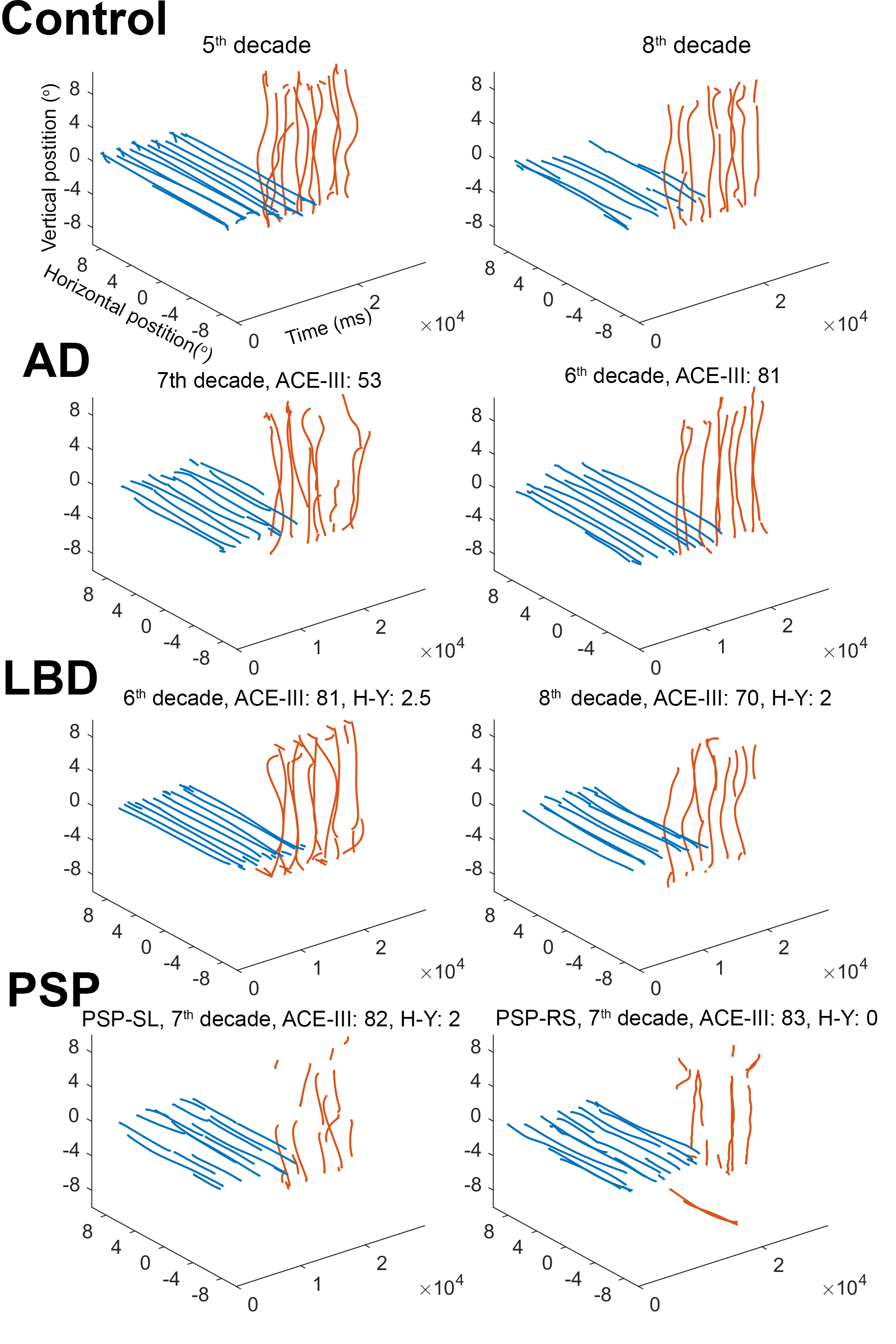
**

*[Caption: Saccade trajectories plotted in horizontal (blue lines), vertical (orange lines) and time axes (ms). (A) Controls: the trajectories from two age-separated controls illustrated that overall saccade trajectories were preserved with age. (B) AD: left, a participant with moderate impairment; right, a participant with mild cognitive impairment (C) LBD: a patient with Parkinson’s disease on the left and one with Dementia with Lewy Body on the right. (D) PSP: PSP-speech/language with mildly saccade velocity declines and PSP-Richardson’s syndrome with markedly velocities reduction. In PSP figures, the vertical and horizontal saccades on the right panel showed wobbling patterns and invariable directional changes compared to one on the left panel.*

**Method: Velocity interruption count, vacillation count and total angular difference**

We computed the velocity and direction following the standardized DEMoNS protocol.^1^

**Method S1. Velocity interruption count**

We computed velocity interruption count from the filtered velocity traces used for saccade detection. Let $v(t)$denote the ^2^eye velocity (in deg/s) sampled at each time point within a single saccade. For each saccade, we scanned through triplets of consecutive velocity samples. A velocity peak was counted whenever the middle sample represented a change in velocity direction, i.e. was either:
  - a local minimum – trough count: $v_{j-2}>v_{j-1}<v_{j}$, or
  - a local maximum – peak count: $v_{j-2}<v_{j-1}>v_{j}$.
The velocity interruption count for that saccade was then defined as the total number of such local extrema:

Velocity interruption count = trough count + peak count

Thus, higher values indicate a more segmented or multiphasic velocity profile (multiple accelerations and decelerations within a single saccade), whereas lower values indicate a more canonical, single-peaked velocity profile.

**Method S2. Vacillation count**

We computed vacillation from the same filtered gaze position traces used for saccade detection. Let $x(t)$ and $y\left( t \right)$denote the horizontal and vertical eye positions (in deg) sampled at each time point within a single saccade. For each axis separately, we identified local extrema by scanning through triplets of consecutive samples. A turning point was counted whenever the middle sample was either:

- a local minimum: $x_{j-2}>x_{j-1}<x_{j}$, or
- a local maximum: $x_{j-2}<x_{j-1}>x_{j}$.

We applied the same criterion to the vertical trace $y(t)$. The total vacillation count for that saccade was then defined as the sum of horizontal and vertical turning points:

$$\text{Vacillation count}=N_{\text{turn,X}}+N_{\text{turn,Y}}.$$

Thus, higher vacillation values indicate that the eye reached the target via multiple direction changes (a more “broken” trajectory), whereas lower values indicate a smoother path.

**Method S3. Sum of direction changes**
For each saccade, the eye position trace was sampled every 1 ms and converted into an instantaneous movement direction, expressed as an angle from 0 - 360° on the polar “clock”. A perfectly straight saccade would have a near-constant direction (e.g. ~180° for a leftward saccade, ~90° for an upward saccade etc.). In practice, however, the direction angle fluctuates over time as the trajectory curves or wobbles.

To quantify this directional instability, we computed the **sum of direction changes**, defined as the sum of the absolute differences between consecutive direction angles along the saccade:

$$\text{Sum of direction changes}=\sum_{i=2}^{N} \mid\theta_{i}-\theta_{i-1}\mid$$

where $\theta_{i}$is the instantaneous direction at sample $i$. For an almost straight saccade, successive angles are similar, and the sum angle difference remains small. For a curved, S-shaped, or waving trajectory, the direction changes more frequently and by larger amounts, producing a higher sum angles difference. Intuitively, this metric corresponds to the magnitude of direction change in one flight.

**Supplementary 1**

**Table S1A.** **Fitting performance of three most common used ‘main sequence’ fitting models for corrected velocity**

| **Model** | | **Akaike information criterion with correction** | **Bayesian information criterion** |
| --- | --- | --- | --- |
| Horizontal | Square root | 200.3 [171.2 - 237.3] | 201.6 [172.3 - 239.2] |
|  | Power law | 199.2 [169.8 - 232.4] | 200.7 [171.0 - 234.8] |
|  | Exponential | 194.3 [168.0 - 232.0] | 196.0 [169.0 - 239.9] |
| Vertical | Square-root | 203.5 [161.7 - 238.2] | 204.6 [162.3 - 237.3] |
|  | Power-law | 203.9 [161.5 - 235.3] | 205.4 [161.8 - 237.3] |
|  | Exponential | 203.6 [159.1 - 237.3] | 204.7 [159.0 - 239.4] |

*Formula:* *Square root formula: V_peak_ = V_corrected_√Amplitude; Power-law: V_peak_ = mAmplitude^K^; Exponential: V_peak_ = E*(1-e^-Amplitude/C^); V_peak_ and Amplitude is the peak velocity and amplitude of saccades used for fitting; V_corrected_, m, K, E, C are constants of each fitting formula. Note that only square root formula has one constant.* *The median with interquartile range of [25 -75] is representing for the average of individual across disorders.*

**Table S1B.** **Fitting performance of three single-parameter formula for velocity interruption**

| **Model** | | **Mean square error** | **Bayesian information criterion** |
| --- | --- | --- | --- |
| Horizontal | Linear | 1.2 [0.9 - 2.1] | 69.8 [56.9 - 86.6] |
|  | Square root | 1.0 [0.6 - 1.7] | 64.5 [51.8 – 80.9] |
|  | Log | 1.0 [0.6 - 1.7] | 64.6 [51.9 - 81.0] |
| Vertical | Linear | 2.2 [1.4 - 3.9] | 77.5 [60.5 - 95.1] |
|  | Square root | 1.9 [1.1 - 3.8] | 74.7 [59.0 - 94.2] |
|  | Log | 2.0 [1.2 - 3.8] | 74.7 [59.7 - 94.4] |

*Formula: Linear model: I_count_ = I*Amplitude; Square root formula: I_count_ = I*√ Amplitude; Log: I_count_ = I*ln(1+Amplitude); I_count_ and Amplitude is vacillation count, and amplitude of saccades used for fitting; I is velocity interruption - a constant of each fitting formula. The median with interquartile range of [25 -75] is representing for the average of individual across disorders.*

**Table S1C.** **Fitting performance of three single-parameter formula for vacillation**

| **Model** | | **Mean square error** | **Bayesian information criterion** |
| --- | --- | --- | --- |
| Horizontal | Linear | 12.1 [9.2 - 18.4] | 115.5 [99.0 - 146.0] |
|  | Square root | 8.0 [5.6 - 11.6] | 106.4 [90.7 - 130.4] |
|  | Log | 8.1 [5.7 - 11.6] | 106.9 [89.9 - 129.2] |
| Vertical | Linear | 12.9 [9.0 - 21.9] | 112.6 [89.8 - 132.9] |
|  | Square root | 9.3 [6.1 - 16.9] | 107.4 [84.4 - 124.5] |
|  | Log | 9.5 [6.4 - 17.2] | 107.3 [84.1 - 124.7] |

*Formula: Linear model: Va_count_ = Va*Amplitude; Square root formula: Va_count_ = Va*√Amplitude; Log: Va_count_ = Va*ln(1+Amplitude); Va_count_ and Amplitude is vacillation count, and amplitude of saccades used for fitting; Va is vacillation - a constant of each fitting formula. The median with interquartile range of [25 -75] is representing for the average of individual across disorders.*

**Table S1D.** **Fitting performance of three single-parameter formula for directional instability**

| **Model** | | **Mean square error** | **Bayesian information criterion** |
| --- | --- | --- | --- |
| Horizontal | Linear | 2205.7 [1565.1 - 3316.7] | 227.5 [190.1 - 262.2] |
|  | Square root | 1338.4 [934.7 - 2000.7] | 215.0 [181.5 - 249.1] |
|  | Log | 1294.8 [936.0 - 2008.0] | 215.0 [180.5 - 248.1] |
| Vertical | Linear | 3220.1 [2233.3 - 4564.5] | 222.0 [180.8 - 260.7] |
|  | Square root | 1976.3 [1269.0 - 2977.4] | 211.0 [170.3 - 247.4] |
|  | Log | 1962.1 [1325.8 - 3065.9] | 210.9 [170.7 - 249.2] |

*Formula: Linear model: SDC = DI*Amplitude; Square root formula: SDC = DI*√ Amplitude; Log: SDC = DI * ln (1+ Amplitude); SDC and Amplitude is angular difference, and amplitude of saccades used for fitting; DI is directional instability - a scaling factor getting from fitting formula. The median with interquartile range of [25 -75] is representing for the average of individual across disorders.*

** Conclusions: Across all novel metrics, square root offered advantageous fitting performance over square root or log formula.*

**Supplementary 2: Corrected velocities**

**Table S2A. Linear mixed model comparing the corrected velocity with fixed effects of diagnosis, direction and the their interactions; with random intercept for each participant**

**Model:** Corrected velocity ~ Diagnosis * Direction + (1|participant)

**Reference group:** Control | Horizontal

| **Variables** | **B** | **SE** | **df** | **t** | **p** |
| --- | --- | --- | --- | --- | --- |
| (Intercept) | 112.96 | 1.65 | 247.6 | 68.61 | < 0.001 |
| Diagnosis | | | | | |
| *AD* | -3.51 | 3.93 | 247.6 | -0.89 | 0.37 |
| *LBD* | -1.03 | 2.79 | 247.6 | -0.37 | 0.71 |
| *PSP* | -20.27 | 3.65 | 247.6 | -5.55 | < 0.001 |
| Direction | | | | | |
| *Vertical* | -9.53 | 1.28 | 184.0 | -7.44 | < 0.001 |
| Interactions | | | | | |
| *AD x Vertical* | 1.12 | 3.06 | 184.0 | 0.37 | 0.71 |
| *LBD x Vertical* | -2.69 | 2.17 | 184.0 | -1.24 | 0.22 |
| *PSP x Vertical* | -4.48 | 2.84 | 184.0 | -1.58 | 0.12 |

B: unstandardized coefficients; SE: standard error; df: degree of freedom.

All assumptions were met.

**Table S2B. Contrast of estimated marginal means of corrected velocities within diagnoses**

| **Contrast of corrected velocities between vertical - horizontal** | **B** | **SE** | **df** | **t ratio** | **p** |
| --- | --- | --- | --- | --- | --- |
| Control | -9.53 | 1.28 | 184 | 7.44 | < 0.001 |
| AD | -8.41 | 2.78 | 184 | 3.03 | 0.003 |
| LBD | -12.21 | 1.76 | 184 | 6.95 | < 0.001 |
| PSP | -14.01 | 2.54 | 184 | 5.53 | < 0.001 |

**Table S2C. Contrast of estimated marginal means of corrected velocities between diagnoses**

| **Contrast of corrected velocities** | | **B** | **SE** | **df** | **t ratio** | **p-value** |
| --- | --- | --- | --- | --- | --- | --- |
| **Horizontal** | Control - AD | 3.51 | 3.93 | 248 | 0.89 | 0.81 |
|  | Control - LBD | 1.02 | 2.79 | 248 | 0.37 | 0.98 |
|  | Control - PSP | 20.27 | 3.65 | 248 | 5.55 | < 0.001 |
|  | AD - LBD | -2.49 | 4.22 | 248 | -0.59 | 0.94 |
|  | AD - PSP | 16.75 | 4.83 | 248 | 3.47 | < 0.01 |
|  | LBD - PSP | 19.24 | 3.96 | 248 | 4.85 | < 0.001 |
| **Vertical** | Control - AD | 2.39 | 3.93 | 248 | 0.61 | 0.93 |
|  | Control - LBD | 3.71 | 2.79 | 248 | 1.33 | 0.55 |
|  | Control - PSP | 24.75 | 3.65 | 248 | 6.78 | < 0.001 |
|  | AD - LBD | 1.32 | 4.22 | 248 | 0.31 | 0.99 |
|  | AD - PSP | 22.36 | 4.83 | 248 | 4.63 | < 0.001 |
|  | LBD - PSP | 21.04 | 3.96 | 248 | 5.31 | < 0.001 |

**Table S2D. Direction-specific z-standardised corrected velocity in PSP**

| **Direction** | **Mean z-score ± SD** | **Normality** | **Paired t-test p-value** | **Cohen’s d** |
| --- | --- | --- | --- | --- |
| Horizontal | -1.43 ± 1.36 | Normal | 0.13 | 0.32 |
| Vertical | -1.72 ± 1.33 | Normal |  |  |

**Supplementary 3: Vertical and horizontal corrected velocities association**

**Table S3. Linear model investigating the vertical and horizontal corrected velocity relationship across diagnoses**

**Model:** Vertical corrected velocity ~ Horizontal corrected velocity * Diagnosis

**Reference group:** Control

| **Variables** | **B** | **SE** | **t** | **p** |
| --- | --- | --- | --- | --- |
| Intercept | 31.74 | 9.67 | 3.28 | 0.001 |
| Horizontal velocity | 0.63 | 0.08 | 7.47 | < 0.001 |
| Diagnosis | | | | |
| *AD* | -19.10 | 17.65 | -1.08 | 0.28 |
| *LBD* | -12.22 | 15.59 | -0.78 | 0.43 |
| *PSP* | -23.63 | 15.32 | -1.54 | 0.12 |
| Interactions | | | | |
| *AD x Horizontal velocity* | 0.17 | 0.16 | 1.10 | 0.27 |
| *LBD x Horizontal velocity* | 0.08 | 0.14 | 0.59 | 0.55 |
| *PSP x Horizontal velocity* | 0.13 | 0.15 | 0.84 | 0.40 |

B: unstandardized coefficients; SE: standard error.

All assumptions were met.

**Supplementary 4: Velocity interruptions**

**TABLE S4A. Linear mixed model comparing the velocity interruption with fixed effects of diagnosis, direction and the their interactions; with random intercept for each participant**

**Best linear model:** Velocity interruption ~ Diagnosis * Direction + (1|participant)

**Reference group:** Control | horizontal

| **Variables** | **B** | **SE** | **df** | **t** | **p** |
| --- | --- | --- | --- | --- | --- |
| Intercept | 0.59 | 0.06 | 282.8 | 10.4 | <0.001 |
| Diagnosis | | | | | |
| *AD* | 0.12 | 0.14 | 282.8 | 0.85 | 0.40 |
| *LBD* | 0.15 | 0.10 | 282.8 | 1.55 | 0.12 |
| *PSP* | 0.81 | 0.13 | 282.8 | 6.40 | <0.001 |
| Direction | | | | | |
| *Vertical* | 0.37 | 0.05 | 184.0 | 6.74 | <0.001 |
| Interactions | | | | | |
| *AD x Vertical* | 0.02 | 0.13 | 184.0 | 0.12 | 0.90 |
| *LBD x Vertical* | 0.09 | 0.09 | 184.0 | 0.94 | 0.35 |
| *PSP x Vertical* | 0.77 | 0.12 | 184.0 | 6.41 | <0.001 |

B: unstandardized coefficients; SE: standard error; df: degree of freedom.

**Table S4B. Contrast of estimated marginal means of vertical - horizontal velocity interruption within diagnoses**

| **Diagnosis** | **B** | **SE** | **df** | **t ratio** | **p** |
| --- | --- | --- | --- | --- | --- |
| Control | 0.37 | 0.05 | 184 | 6.74 | <0.001 |
| AD | 0.38 | 0.12 | 184 | 3.24 | 0.001 |
| LBD | 0.45 | 0.07 | 184 | 6.08 | <0.001 |
| PSP | 1.14 | 0.11 | 184 | 10.59 | <0.001 |

**Table S4C. Contrast of estimated marginal means of velocity interruption between diagnoses**

| **Diagnosis** | | **B** | **SE** | **df** | **t ratio** | **p-value** |
| --- | --- | --- | --- | --- | --- | --- |
| **Horizontal** | Control - AD | -0.12 | 0.14 | 283 | -0.86 | 0.83 |
|  | Control - LBD | -0.15 | 0.10 | 283 | -1.55 | 0.41 |
|  | Control - PSP | -0.81 | 0.13 | 283 | -6.40 | <0.001 |
|  | AD - LBD | -0.03 | 0.15 | 283 | -0.23 | 1.00 |
|  | AD - PSP | -0.69 | 0.17 | 283 | -4.14 | <0.001 |
|  | LBD - PSP | -0.66 | 0.14 | 283 | -4.80 | <0.001 |
| **Vertical** | Control - AD | -0.13 | 0.14 | 283 | -0.97 | 0.77 |
|  | Control - LBD | -0.24 | 0.10 | 283 | -2.44 | 0.07 |
|  | Control - PSP | -1.58 | 0.13 | 283 | -12.49 | <0.001 |
|  | AD - LBD | -0.10 | 0.15 | 283 | -0.72 | 0.89 |
|  | AD - PSP | -1.45 | 0.17 | 283 | -8.65 | <0.001 |
|  | LBD - PSP | -1.34 | 0.14 | 283 | -9.78 | <0.001 |

**Table S4D. Direction-specific z-standardised velocity interruption in PSP**

| **Direction** | **Median z-score, IQR [25 – 75 ]** | **Normality** | **Wilcoxon p-value** | **Effect size**  **r** |
| --- | --- | --- | --- | --- |
| Horizontal | 3.43 [1.70 - 5.31] | Non-Normal | 0.66 | 0.035 |
| Vertical | 4.38 [1.43 - 7.11] | Non-Normal |  |  |

**Supplementary 5: Vacillation**

**Table S5A.** **Linear mixed effect model to predict vacillation basing on fixed effects: diagnosis, direction and their interaction and random effect: individual participant.**

**Model:** Vacillation ~ Diagnosis * Direction + (1|participant)

**Reference group:** Horizontal | Control.

| **Variables** | **B** | **SE** | **df** | **t** | **p** |
| --- | --- | --- | --- | --- | --- |
| Intercept | 2.65 | 0.08 | 93 | 33.90 | < 0.001 |
| Diagnosis | | | | | |
| *AD* | 0.43 | 0.19 | 27.3 | 2.26 | 0.03 |
| *LBD* | 0.48 | 0.18 | 99.7 | 2.68 | 0.01 |
| *PSP* | 2.27 | 0.49 | 35.2 | 4.65 | <0.001 |
| Direction | | | | | |
| *Vertical* | 0.13 | 0.07 | 93.0 | 2.02 | 0.04 |
| Interactions | | | | | |
| *AD x Vertical* *vacillation* | -0.03 | 0.20 | 27.3 | -0.14 | 0.89 |
| *LBD x Vertical* *vacillation* | 0.10 | 0.17 | 99.7 | 0.60 | 0.55 |
| *PSP x Vertical* *vacillation* | 0.82 | 0.36 | 35.2 | 2.30 | 0.03 |

*Note:* B: unstandardized coefficients; SE: standard error; df: degree of freedom.

This model showed the heteroskedasticity, so the cluster robust type-3 was used to estimate the standard error and p-value more conservatively than conventional method.

**Table S5B.** **Contrast of estimated marginal means of vertical – horizontal vacillation within diagnoses.**

| **Diagnosis** | **B** | **SE** | **z ratio** | **p** |
| --- | --- | --- | --- | --- |
| Control | 0.13 | 0.07 | 2.02 | 0.04 |
| AD | 0.10 | 0.19 | 0.56 | 0.58 |
| LBD | 0.23 | 0.15 | 1.51 | 0.13 |
| PSP | 0.96 | 0.35 | 2.71 | 0.01 |

* Degrees-of-freedom method: asymptotic

**Table S5C.** **Contrast of estimated marginal means of vacillation between diagnoses**

| **Diagnosis** | | **B** | **SE** | **z ratio** | **p** |
| --- | --- | --- | --- | --- | --- |
| Horizontal | Control - AD | -0.43 | 0.19 | -2.26 | 0.11 |
|  | Control - LBD | -0.48 | 0.18 | -2.68 | 0.04 |
|  | Control - PSP | -2.27 | 0.49 | -4.65 | < 0.001 |
|  | AD - LBD | -0.04 | 0.24 | -0.18 | 1.00 |
|  | AD - PSP | -1.84 | 0.51 | -3.58 | 0.002 |
|  | LBD - PSP | -1.79 | 0.51 | -3.53 | 0.002 |
| Vertical | Control - AD | -0.41 | 0.26 | -1.59 | 0.38 |
|  | Control - LBD | -0.58 | 0.19 | -3.03 | 0.01 |
|  | Control - PSP | -3.09 | 0.51 | -6.05 | < 0.001 |
|  | AD - LBD | -0.17 | 0.29 | -0.59 | 0.94 |
|  | AD - PSP | -2.69 | 0.56 | -4.83 | < 0.001 |
|  | LBD - PSP | -2.52 | 0.53 | -4.76 | < 0.001 |

* Degrees-of-freedom method: asymptotic

**Table 5D. Direction-specific z-standardised vacillation in PSP.**

| **Direction** | **Median z-score, IQR [25 – 75 ]** | **Normality** | **p-value** | **Effect size r** |
| --- | --- | --- | --- | --- |
| Horizontal | 1.80, [1.32 – 3.73] | Non-normal | 0.32 | 0.175 |
| Vertical | 2.98, [1.50 – 5.46] | Non-normal |  |  |

**Supplementary 6: Directional instability**

**TABLE S6A. Linear mixed model comparing the directional instability with fixed effects of diagnosis, direction and the their interactions; with random intercept for each participant**

**Model**: Directional instability ~ Diagnosis * Direction + Mean-centered age + (1 | participant)

Reference group: Control | Horizontal | Mean age of 67.14

| **Variables** | **B** | **SE** | **t** | **df** | **p** |
| --- | --- | --- | --- | --- | --- |
| (Intercept) | 22.73 | 0.56 | 40.51 | 91.6 | <0.001 |
| Diagnosis | | | | | |
| *AD* | 6.03 | 2.68 | 2.25 | 27.3 | 0.03 |
| *LBD* | 7.78 | 2.21 | 3.51 | 93.7 | <0.001 |
| *PSP* | 28.97 | 5.13 | 5.65 | 35.6 | <0.001 |
| Mean-centred age | 0.22 | 0.13 | 1.70 | 58.4 | 0.09 |
| Direction | | | | | |
| *Vertical* | 14.68 | 0.9 | 16.31 | 93.0 | <0.001 |
| Interactions | | | | | |
| *AD x Vertical* | 0.51 | 2.79 | 0.18 | 27.3 | 0.86 |
| *LBD x Vertical* | 1.00 | 2.25 | 0.44 | 99.7 | 0.66 |
| *PSP x Vertical* | 21.86 | 5.70 | 3.84 | 35.2 | < 0.001 |

B: unstandardized coefficients; SE: standard error; df: degree of freedom.

This model showed the heteroskedasticity, so the cluster robust type-3 was used to estimate the standard error and p-value more conservatively than conventional method.

**Table S6B.** **Contrast of estimated marginal means of vertical - horizontal directional instability within diagnoses.**

| **Diagnosis** | **B** | **SE** | **z ratio** | **p** |
| --- | --- | --- | --- | --- |
| Control | 14.68 | 0.9 | 16.31 | < 0.001 |
| AD | 15.19 | 2.64 | 5.75 | < 0.001 |
| LBD | 15.68 | 2.06 | 7.60 | < 0.001 |
| PSP | 36.54 | 5.63 | 6.50 | < 0.001 |

* Degrees-of-freedom method: asymptotic

**Table S6C.** **Contrast of estimated marginal means of directional instability between diagnoses.**

| **Diagnosis** | | **B** | **SE** | **z ratio** | **p** |
| --- | --- | --- | --- | --- | --- |
| **Horizontal** | Control - AD | -6.03 | 2.68 | -2.25 | 0.32 |
|  | Control - LBD | -7.71 | 2.22 | -3.48 | 0.01 |
|  | Control - PSP | -28.97 | 5.13 | -5.65 | < 0.001 |
|  | AD - LBD | -1.69 | 3.53 | -0.48 | 1.00 |
|  | AD - PSP | -22.94 | 5.78 | -3.97 | <0.01 |
|  | LBD - PSP | -21.19 | 5.51 | -3.84 | <0.01 |
| **Vertical** | Control - AD | -6.54 | 2.90 | -2.25 | 0.32 |
|  | Control - LBD | -8.71 | 2.19 | -3.98 | 0.001 |
|  | Control - PSP | -50.83 | 6.36 | -7.99 | < 0.001 |
|  | AD – LBD | -2.17 | 3.58 | -0.61 | 1.00 |
|  | AD - PSP | -44.29 | 6.94 | -6.38 | < 0.001 |
|  | LBD - PSP | -42.05 | 6.69 | -6.29 | < 0.001 |

* Degrees-of-freedom method: asymptotic

**Table 6D. Direction-specific z-standardised of directional instability in PSP.**

| **Saccade direction** | **Median z-score, IQR [25 – 75 ]** | **Normality** | **Wilcoxon test**  **p-value** | **Effect size**  **r** |
| --- | --- | --- | --- | --- |
| Horizontal | 5.26, [2.58 - 8.64] | Normal | 0.65 | 0.023 |
| Vertical | 5.76, [3.61 - 8.45] | Non-normal |  |  |

**Table S7. Sensitivity analysis of PSP subgroup (PSP-RS and PSP-nonRS) cross metrics**

| Comparison | PSP-RS  (n = 10) | PSP-non-RS  (n = 14) | Omnibus  p-value | p_FDR_ | Control  reference |
| --- | --- | --- | --- | --- | --- |
| Horizontal corrected velocity | 87.3 ± 21.9 | 96.6 ± 16.9 | 0.28 | 0.28 | 113.0 ± 14.2 |
| Vertical corrected velocity | 69.3 ± 18.3 | 85.4 ± 17.1 | 0.04 | 0.13 | 103.4 ± 14.4 |
| Horizontal velocity interruption | 1.35  [0.94 – 2.31] | 1.17  [0.78 – 1.33] | 0.21 | 0.28* | 0.6 [0.4 - 0.7] |
| Vertical velocity interruption | 2.7  [2.4 – 3.4] | 1.6  [1.2 – 2.7] | 0.07 | 0.13* | 0.9 [0.8 – 1.1] |
| Horizontal vacillation | 5.8 ± 3.1 | 4.3 ± 1.3 | 0.19 | 0.27 | 2.7 ± 0.8 |
| Vertical vacillation | 7.1 ± 2.5 | 5.0 ± 2.1 | 0.05 | 0.13 | 2.8 ± 0.9 |
| Horizontal directional instability | 63.8 ± 29.0 | 43.3 ± 16.9 | 0.06 | 0.13 | 22.3 ± 4.6 |
| Vertical directional instability | 97.1 ± 30.7 | 82.1 ± 29.9 | 0.25 | 0.27 | 36.9 ± 7.9 |

(*) Wilcoxon signed rank tests were used.

**Table S8. Correlation table between disease stage and oculomotor metrics in PSP**

| Disease stage | Oculomotor metrics | Spearman rho | P value | P_FDR_ |
| --- | --- | --- | --- | --- |
| H – Y scale | Horizontal corrected velocity | -0.03 | 0.89 | 0.91 |
|  | Vertical corrected velocity | -0.03 | 0.90 | 0.91 |
|  | Horizontal velocity interruption | 0.02 | 0.91 | 0.91 |
|  | Vertical velocity interruption | -0.14 | 0.51 | 0.88 |
|  | Horizontal vacillation | -0.13 | 0.54 | 0.88 |
|  | Vertical vacillation | -0.07 | 0.73 | 0.91 |
|  | Horizontal directional instability | 0.13 | 0.55 | 0.88 |
|  | Vertical directional instability | -0.02 | 0.91 | 0.91 |
| Disease duration | Horizontal corrected velocity | -0.20 | 0.34 | 0.88 |
|  | Vertical corrected velocity | -0.25 | 0.24 | 0.88 |
|  | Horizontal velocity interruption | 0.14 | 0.51 | 0.88 |
|  | Vertical velocity interruption | 0.30 | 0.15 | 0.80 |
|  | Horizontal vacillation | 0.05 | 0.83 | 0.91 |
|  | Vertical vacillation | 0.35 | 0.09 | 0.72 |
|  | Horizontal directional instability | 0.15 | 0.50 | 0.88 |
|  | Vertical directional instability | 0.37 | 0.08 | 0.72 |

**References:**

1. Nij Bijvank JA, Petzold A, Balk LJ, et al. A standardized protocol for quantification of saccadic eye movements: DEMoNS. PLoS One. 2018;13(7):e0200695.

2. Hoglinger GU, Respondek G, Stamelou M, et al. Clinical diagnosis of progressive supranuclear palsy: The movement disorder society criteria. Mov Disord. 2017 Jun;32(6):853-64.
